# Disulfiram Produces Potent Anxiolytic-Like Effects Without Benzodiazepine Anxiolytics-Related Adverse Effects in Mice

**DOI:** 10.3389/fphar.2022.826783

**Published:** 2022-03-07

**Authors:** Akiyoshi Saitoh, Yoshifumi Nagayama, Daisuke Yamada, Kosho Makino, Toshinori Yoshioka, Nanami Yamanaka, Momoka Nakatani, Yoshino Takahashi, Mayuna Yamazaki, Chihiro Shigemoto, Misaki Ohashi, Kotaro Okano, Tomoki Omata, Etsuko Toda, Yoshitake Sano, Hideyo Takahashi, Kouji Matsushima, Yuya Terashima

**Affiliations:** ^1^ Laboratory of Pharmacology, Faculty of Pharmaceutical Sciences, Tokyo University of Science, Chiba, Japan; ^2^ Department of Medicinal Chemistry, Faculty of Pharmaceutical Sciences, Tokyo University of Science, Chiba, Japan; ^3^ Division of Molecular Regulation of Inflammatory and Immune Diseases, Research Institute for Biomedical Sciences (RIBS), Tokyo University of Science, Chiba, Japan; ^4^ Department of Analytic Human Pathology, Nippon Medical School, Tokyo, Japan; ^5^ Department of Applied Biological Science, Faculty of Science and Technology, Tokyo University of Science, Chiba, Japan

**Keywords:** anxiolytic, anxiety, FROUNT, frontal cortex, alcoholism, chemokine

## Abstract

Disulfiram is an FDA approved drug for the treatment of alcoholism. The drug acts by inhibiting aldehyde dehydrogenase, an enzyme essential to alcohol metabolism. However, a recent study has demonstrated that disulfiram also potently inhibits the cytoplasmic protein FROUNT, a common regulator of chemokine receptor CCR2 and CCR5 signaling. Several studies have reported that chemokine receptors are associated with the regulation of emotional behaviors in rodents, such as anxiety. Therefore, this study was performed to clarify the effect of disulfiram on emotional behavior in rodents. The anxiolytic-like effects of disulfiram were investigated using an elevated plus-maze (EPM) test, a typical screening model for anxiolytics. Disulfiram (40 or 80 mg/kg) significantly increased the amount of time spent in the open arms of the maze and the number of open arm entries without affecting the total open arms entries. Similar results were obtained in mice treated with a selective FROUNT inhibitor, disulfiram-41 (10 mg/kg). These disulfiram-associated behavioral changes were similar to those observed following treatment with the benzodiazepine anxiolytic diazepam (1.5 mg/kg). Moreover, disulfiram (40 mg/kg) significantly and completely attenuated increased extracellular glutamate levels in the prelimbic-prefrontal cortex (PL-PFC) during stress exposure on the elevated open-platform. However, no effect in the EPM test was seen following administration of the selective aldehyde dehydrogenase inhibitor cyanamide (40 mg/kg). In contrast to diazepam, disulfiram caused no sedation effects in the open-field, coordination disorder on a rotarod, or amnesia in a Y-maze. This is the first report suggesting that disulfiram produces anxiolytic-like effects in rodents. We found that the presynaptic inhibitory effects on glutaminergic neurons in the PL-PFC may be involved in its underlying mechanism. Disulfiram could therefore be an effective and novel anxiolytic drug that does not produce benzodiazepine-related adverse effects, such as amnesia, coordination disorder, or sedation, as found with diazepam. We propose that the inhibitory activity of disulfiram against FROUNT function provides an effective therapeutic option in anxiety.

## 1 Introduction

Disulfiram (DSF), a disulfide derivative of N,N-diethyl dithiocarbamate, is an FDA approved drug for the treatment of alcoholism. During the 19th century, employees in the rubber manufacturing industry who were exposed to DSF at work suffered severe adverse effects to alcohol and began to avoid the consumption of alcoholic beverages. Later, these effects were found to be caused by DSF’s inhibition of aldehyde dehydrogenase (ALDH), an enzyme essential to alcohol metabolism (Chen et al., 2006). Today, DSF is used as a useful treatment option for those suffering from alcoholism (Skinner et al., 2014).

Recently, it was found that DSF also potently inhibits the cytoplasmic protein FROUNT (Table 1) (Terashima et al., 2020), a common regulator of chemokine receptor CCR2 and CCR5 signaling, which controls the directional migration that facilitates monocyte/macrophage infiltration (Terashima et al., 2005; Toda et al., 2009; Toda et al., 2014). Interestingly, multistep screening from a library of 131,200 compounds highlighted that DSF blocks FROUNT interaction with CCR2 and CCR5 by direct binding to a specific site in the chemokine receptor-binding domain of the FROUNT protein (Terashima et al., 2020). In addition, synergistic treatment with DSF and an immune checkpoint antibody was found to inhibit tumor growth in a mouse model. Now, DSF is being investigated in combination with the anti-PD-1 antibody in patients with gastric cancer (jRCTs031180183).

**TABLE 1:** Inhibition properties of DSF, DSF-41, and cyanamide.

IC_50_	DSF	DSF-41	Cyanamide
Structure	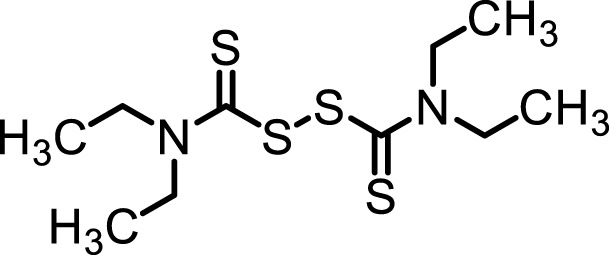	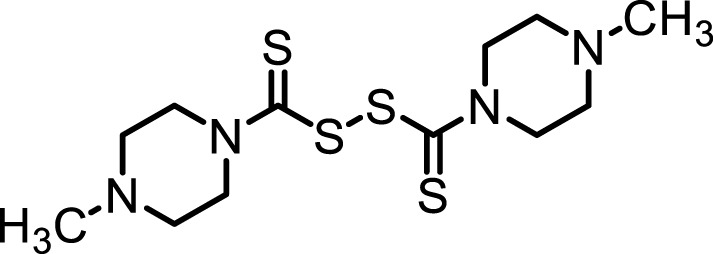	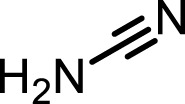
FROUNT/CCR2	258 nM	62 nM	1,000 nM<

IC_50_ values from inhibition experiments with DSF, its derivative DSF-41, and cyanamide, a selective inhibitor of aldehyde dehydrogenase (ALDH).

Although the functions of FROUNT-chemokines signaling in the immune system are well documented, the potential role of CNS-expressed FROUNT-chemokine-related molecules as neuromodulators remains largely unknown. Several studies have reported that chemokine receptors are associated with the regulation of emotional behaviors, such as anxiety, in rodents (Rostène et al., 2007; Schroeder et al., 2010; Milenkovic et al., 2019). Chemokine receptor knockout mice are reported to display depressive-like and fear/anxiety-like behaviors (Jaehne and Baune, 2014; Ambrée et al., 2016; Choi et al., 2018). Although many studies on the anti-drug abuse effects of DSF, such as attenuating cocaine craving (Gaval-Cruz and Weinshenker, 2009; Schroeder et al., 2010) or alcohol consumption (Tampier et al., 2008; Gaval-Cruz and Weinshenker, 2009), have been reported in preclinical studies, there have been no studies on the emotional behaviors related to FROUNT signaling and DSF. In this study, we coincidentally discovered the anxiolytic-like effects of DSF while evaluating the secondary pharmacological properties of the drug in a preclinical study. Interestingly, we found that a more potent novel FROUNT inhibitor DSF-41 showed anxiolytic-like effects at lower doses than DSF (Table 1), and these effects were different to those induced by another alcohol deterrent drug cyanamide, which is a more selective ALDH inhibitor than DSF.

Benzodiazepines are widely used as anxiolytics. This group of drugs has low toxicity, but it has sedative, hypnotic, and muscle relaxant side effects. The benzodiazepines also impair cognitive performance and memory, while adversely affecting motor control. The anxiolytic effects of this class of drugs are mediated by allosteric interactions with the benzodiazepine-GABA_A_ receptor complex. Thus, efforts to overcome some of the unwanted effects have led to the development of novel anxiolytics that act independently from the mechanisms of the benzodiazepine-GABA_A_ receptor complex. Here we report that DSF produces robust anxiolytic-like effects without producing benzodiazepine anxiolytic-related adverse effects in animal models.

## 2 Materials and Methods

### 2.1 Animals

Male ICR mice were used for behavioral experiments (age: 6–10 weeks; Tokyo Laboratory Animals Science Co., Ltd., Tokyo, Japan). The total number of animals used in the study were 232 animals. All animals had free access to food and water in an animal room maintained at 23 ± 1°C with a 12 h light–dark cycle (lights were automatically switched on at 8:00 a.m.). Animals were housed in transparent plastic cages (W225 ×D338 × H140 mm), with six animals per cage. The study was conducted in accordance with protocols approved by the Institutional Animal Care and Use Committee of the Tokyo University of Science (approval no. Y18063).

### 2.2 Drugs

The drugs used in the present study were DSF (Mitsubishi Tanabe Pharma Corporation, Osaka, Japan), cyanamide (Wako Pure chemical Industries, Ltd. Tokyo, Japan), DSF-41 (synthesized by Takahashi et al., Tokyo university of Science, Tokyo, Japan), and diazepam (Wako Pure chemical Industries, Ltd. Tokyo, Japan). DSF, cyanamide, and DSF-41 were dissolved in 2% Tween 80 (MP Biomedicates LLC)/10% DMSO (Wako Pure chemical Industries, Ltd. Tokyo, Japan)/88% corn oil (Wako Pure chemical Industries, Ltd. Tokyo, Japan). Diazepam was dissolved in 4.5% hydroxypropyl-*β*-cyclodextrin (Wako Pure chemical Industries, Ltd. Tokyo, Japan). DSF, cyanamide, DSF-41, or vehicle was administered at a volume of 12.5 ml/kg of body weight. Diazepam or its vehicle was administered at a volume of 10 ml/kg of body weight. The administration volume of DSF-41 was identical to that of DSF to enable comparison. The volume of diazepam to be administered was decided on the basis of our previous report (Saitoh et al., 2013).

### 2.3 Elevated Plus-Maze Test

The elevated plus-maze (EPM) test was performed using a modification to the procedure described in our previous report (Saitoh et al., 2013). The EPM apparatus consisted of four arms set in a cross pattern from a neutral central square. Vertical walls (closed arms, 30 cm × 6 cm × 20 cm) delimited two opposing arms, whereas the two other opposing arms had unprotected edges (open arms, 30 cm × 6 cm). The maze was elevated 40 cm above the floor and placed in indirect light (150–200 Lux). Animals were kept in the experimental room for at least 1 h for adaptation before drug administration. DSF (40 mg/kg, i. p.) was administered to animals 15 min (n = 9), 30 min (n = 8), 60 min (*n* = 8), and 120 min (*n* = 8) before the EPM test. The doses of DSF (Terashima et al., 2020) and time schedule (Saitoh et al., 2013) were optimized during our previous studies that used the EPM test. Diazepam (1.5 mg/kg, s. c.) was used as a positive control anxiolytic drug and administered 30 min before the EPM test. The doses of diazepam (Saitoh et al., 2013) were determined during our previous studies that used the EPM test. At the beginning of the 5 min test session, each mouse was placed in the central neutral zone, facing one of the closed arms. The total number of visits to the closed and open arms and the cumulative time spent (percentage of time spent in an open arm) and visits to the open arms (percentage of open arm entries) were counted on a monitor using a video camera system. An arm visit was recorded when the mouse moved its hindfeet into the arm. After the removal of each animal, the apparatus was cleaned with 70% ethanol.

### 2.4 Measurement of Locomotor Activity

The locomotor activity test was performed using a modification to procedures described previously (Yamada et al., 2019). Locomotor activity was monitored using an activity sensor unit for mice (Supermex PAT. P, Muromachi Kikai Co., Ltd.). Each mouse was placed into an apparatus (W225 ×D338 × H140 mm) located beneath fluorescent light (100–400 Lux). Locomotor activity was measured for 30 and 210 min after drug administration and data were analyzed to determine the distance traveled for 3 h. The apparatus was cleaned with 70% ethanol after every test.

### 2.5 Y-Maze Test

The Y-maze test was performed using a modification to procedures described previously (Saitoh et al., 2013). The Y-maze apparatus consisted of three arms positioned at equal angles. Each arm was 40 cm long, with walls 12 cm high and 3 cm wide, and illuminated by indirect light (60–70 Lux). Animals were kept in the experimental room for at least 1 h to adapt before drug administration. DSF (80 mg/kg, i. p.) and diazepam (2.0 mg/kg, s. c.) were administered 30 min before the Y-maze test. The dose of diazepam (Saitoh et al., 2013) was determined during our previous studies that used the EPM test. At the beginning of the 8 min test session, each mouse was placed at the end of one arm. An arm visit was recorded when the mouse moved its hindfeet into the arm. The total number of visits to the three arms was then counted on a monitor using a video camera system. After the test period, we calculated the percentage of alternations according to the following formula [(actual alternations/maximum possible alternations) × 100]. Mice for which the total number of arm entries was <19 were excluded from the analysis (2/39 mice) because these mice did not show adequate exploratory behaviors in the Y-maze device as well as showed insufficient memory evaluation. After the removal of each animal, the apparatus was cleaned with 70% ethanol.

### 2.6 Rotarod

Motor coordination and balance were evaluated using an accelerating rotarod (RTR-R1, MELQUEST Co., Ltd., Toyama, Japan) and illuminated by indirect light (60–70 Lux). Prior to the experiments, the animals were trained to remain for 180 s on a bar, which was 2.5 cm in diameter and 25 cm above the floor and rotated at a speed of 10 rpm. The riding ability of the animals on the rotarod was verified prior to the experiment. As a training session, 1 day before the test, animals were kept in the experimental room for at least 1 h for adaptation and then placed on the bar that was rotating at 10 rpm. The number of falls and time spent on the rotating bar were assessed for a period of 180 s (Chen et al., 2018). Conditions of the training session were as described previously (Hosseinzadeh et al., 2016), and thus, mice that fell more than seven times (2 out of 41 mice) were removed from the training session. In the rotarod test, mice that could remain on the rotating bar for a certain duration without falling off (180 s) during a training session were evaluated for coordination motor disorder after drug administration in subsequent testing sessions. In the test session, animals were kept in the experimental room for at least 1 h for adaptation prior to drug administration. DSF (80 mg/kg, i. p.) and diazepam (1.5, 2.0 mg/kg, s. c.) were administered 30 min before the rotarod test. The dose of diazepam was optimized in a previous study (Saitoh et al., 2013), and the apparatus was wiped clean with 70% ethanol after each test/trial.

### 2.7 Quantification of Brain Noradrenalin and the Concentration of its Metabolite

The quantification of noradrenalin was conducted in accordance with our previously described methods (Takeuchi et al., 2020). Mice were decapitated 30 min after the administration of DSF (80 mg/kg, i. p.) or fusaric acid (100 mg/kg, i. p.). Fusaric acid was used as positive control and the dose was determined during previous studies (Vetulani et al., 1973). Immediately after decapitation, the prefrontal cortex (PFC), amygdala, striatum, and hypothalamus were quickly dissected and placed on an ice-cold glass plate. The brain tissues were stored at −80°C until use.

High-performance liquid chromatography (HPLC) was used to quantify noradrenaline and its metabolite 3-methoxy-4-hydroxyphenylethyleneglycol (MHPG) in four brain regions (PFC, amygdala, striatum, and hypothalamus). The brain tissues were homogenized in 350 μl of 0.2 M perchloric acid containing 100 ng of isoproterenol, which was added as an internal standard. To remove the proteins completely, the homogenates were placed in cold water for 30 min and then centrifuged at 15,000 g for 15 min at 0°C. The upper layer was maintained at pH 3.0 using 1 M sodium acetate. Ten microliter samples were analyzed by HPLC with electrochemical detection. The electrochemical detector (ECD-300; EICOM Co., Kyoto, Japan) was equipped with a graphite electrode (WE-3G; EICOM Co., Kyoto, Japan) that was used at a voltage setting of 750 mV with an Ag/AgCl reference electrode. The mobile phase consisted of a 0.1 M sodium acetate/0.1 M citric acid buffer (pH 3.5) containing 12% methanol, 120 mg/L sodium 1-octanesulfonate (SOS), and 5 mg/L EDTA-2 Na. Monoamines were separated on a C-18 column (150 mm × 3 mm reverse-phase, EICOMPAK SC- 5ODS, EICOM Co.). The mobile phase flow rate was maintained at 0.3 ml/min with a column temperature of 30.0°C.

### 2.8 Microanalysis Study

Mice were anesthetized with sodium pentobarbital (50 mg/kg, i. p.) and placed in a stereotactic apparatus (Kopf 900; David Kopf Instruments, Tujunga, CA). A stainless steel microdialysis guide cannula (outer diameter: 0.37 mm) was implanted into the pre-limbic prefrontal cortex (PL-PFC; AP 1.98 mm, ML 0.3 mm, DV 0.9 mm from bregma). The guide cannula was secured to the skull using a bone anchor screw and dental acrylic cement. Postoperatively, animals were housed in single cages and allowed to recover from surgery for at least 24 h before the microdialysis experiments. Microdialysis probes (A-I-3-01; EICOM, Kyoto, Japan) with 1.0 mm-long membranes were continuously perfused with medium at a flux rate of 2.0 ml/min using a gas-tight syringe pump (ESP-64; EICOM). Immediately after probe insertion, each mouse was attached to a swivel unit (TSU-20; EICOM) to allow free movement in the cage. Microdialysis sampling started 60 min after the onset of probe perfusion. Samples of dialysate were collected every 10 min for 60 min to determine the basal levels of glutamate. After these were determined, DSF (40 mg/kg, i. p.) was administered. The mice were exposed to elevated open-platform stress (Miyata et al., 2007b) 30 min after drug administration and returned to their cages following 30 min of exposure to the stress. The elevated open-platform stress was conducted in a room illuminated with white fluorescent light (300 lux) wherein a transparent acrylic cylinder (11.4 cm diameter, 18 cm-high) was positioned upside-down and mice were placed individually on the top (open-platform). Dialysate samples were then collected for 10 min. The location of the dialysis probes was verified at the end of each experiment in 40 μm-thick brain slices.

The quantification of glutamate was conducted in accordance with our previously described methods (Sakamoto et al., 2021). The glutamate content of the samples was analyzed by HPLC using an electrochemical detector (HTEC-700; EICOM). Briefly, a reverse-phase column (Eicompack GU-GEL, 4.6 3 150 mm; EICOM) was used for separation and a glutamate oxidase immobilized reactor column (E-ENZYMPAK, 3 3 4 mm; EICOM) was used for the conversion of glutamate to hydrogen peroxide at 33°C. The potential of the platinum electrode (WE-PT; EICOM) was set at 10.45 V (vs. Ag/AgCl). The composition of the mobile phase for the measurement of glutamate was 60 mM ammonium chloride buffer (pH 7.6) with 0.7 mM hexadecyl-trimethyl-ammonium bromide (Nacalai Tesque, Kyoto, Japan).

### 2.9 Data Analysis

Data are expressed as the mean ± S.E.M. One-way factorial analysis of variance (ANOVA) was used to compare more than two groups. The statistical significance of the differences in the microdialysis data was assessed by two-way repeated measures ANOVA with interaction (time × drug treatment). *Post-hoc* individual group comparisons were made using Bonferroni’s test for multiple comparisons. Student’s *t*-test was used for comparisons between two groups. All statistical analyses were performed using Graphpad Prism^®^ (Graphpad^®^ Software Inc., San Diego, CA, United States). *p*-values less than 0.05 were considered significant.

## 3 Results

### 3.1 Elevated Plus-Maze Test

#### 3.1.1 The Effect of DSF and Diazepam on the Percentage of Time Spent in and Entries to Open Arms

**FIGURE 1:**
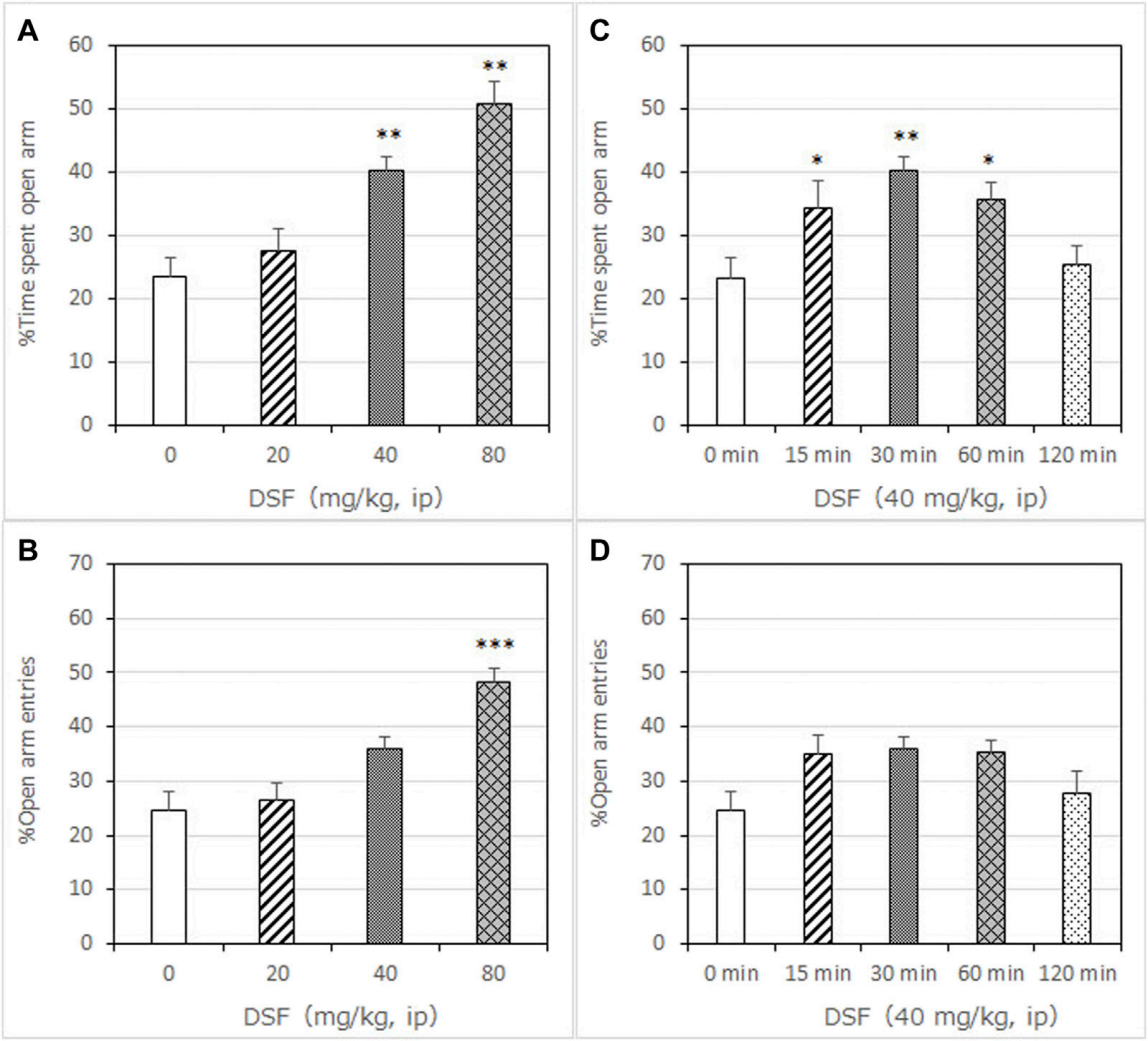
Anxiolytic-like effects of DSF in a mouse elevated plus-maze test. Panel **(A)** shows the percentage of time spent in the open arms. Panel **(B)** shows the percentage of entries into the open arms. DSF and vehicle were administered 30 min before the test. The columns represent the following in order: Vehicle (*n* = 9); DSF 20 mg/kg (*n* = 8), 40 mg/kg (*n* = 8), and 80 mg/kg (*n* = 9). Thirty-four mice were used in total. Statistical significance is denoted by **p < 0.01 and ***p < 0.001, respectively, vs. vehicle-treated mice. Panels **(C,D)** show the percentage of time spent in the open arms **(C)** and entries into the open arms **(D)** following DSF (40 mg/kg, i. p.) administration. DSF was administered 15, 30, 60, and 120 min before the test. The columns represent the following in order: Vehicle (*n* = 9); DSF 15 min (*n* = 9), 30 min (*n* = 8), 60 min (*n* = 8), and 120 min (*n* = 8). Each column represents the mean ± SEM. The statistical significance of differences among the DSF treatment groups was assessed using one-way ANOVA. Post-hoc individual group comparisons were made with Bonferroni’s test. Statistical significance is denoted by **p < 0.01 and ***p < 0.001, respectively, vs. vehicle-treated mice.

**FIGURE 2:**
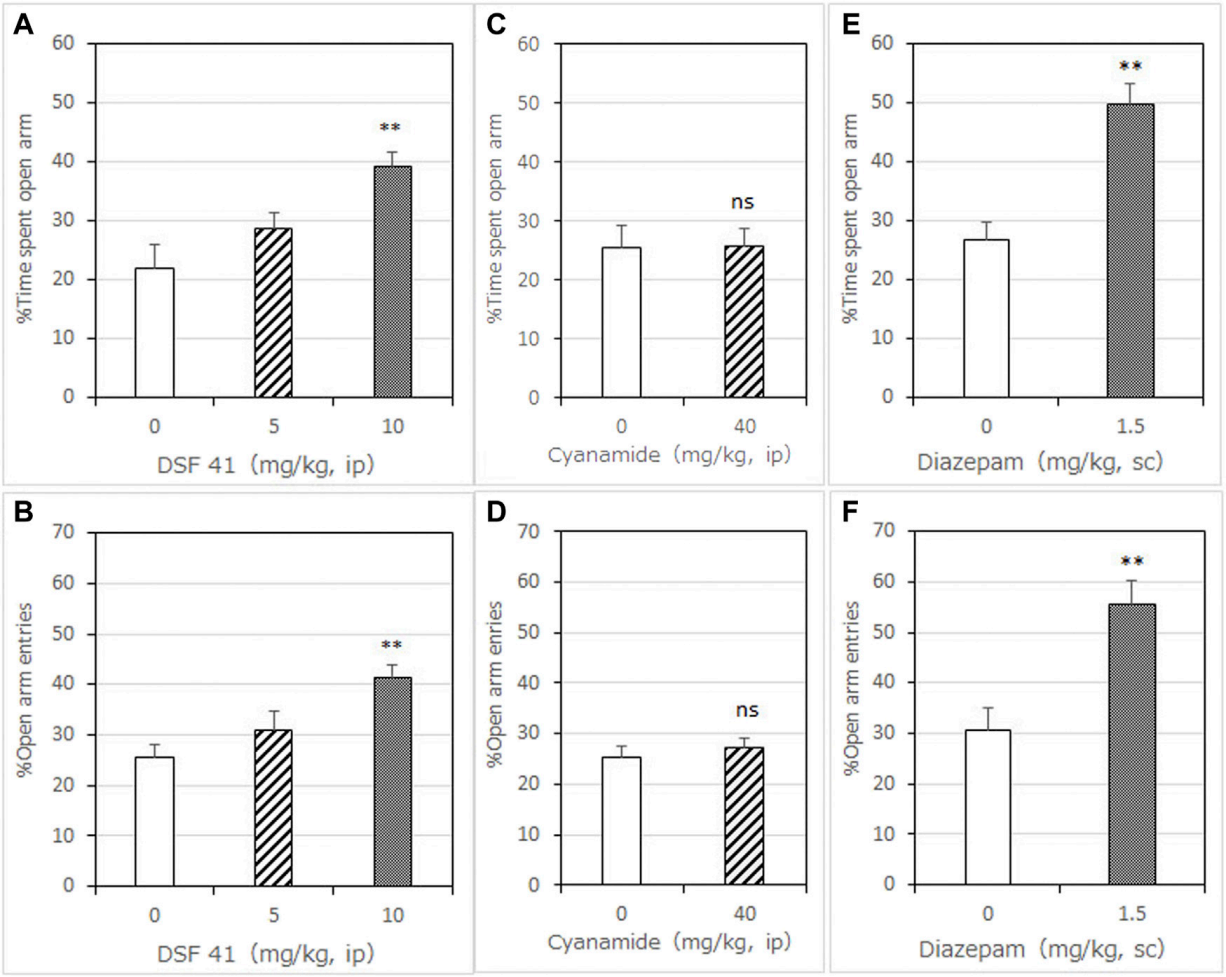
Anxiolytic-like effects of DSF-41, cyanamide, and diazepam in the mouse elevated plus-maze test. Panels **(A,C,E)** show the percentage of time spent in the open arms. Panels **(B,D,F)** show the percentage of entries into the open arms. Diazepam was used as an anxiolytic positive control drug. Drugs were administered 30 min before the test. Each column represents the mean ± SEM. The statistical significance of differences among the DSF-41 treatment groups was assessed using one-way ANOVA. Post-hoc individual group comparisons were made with Bonferroni’s test. The statistical analysis for the cyanamide and diazepam experiments was performed using Student’s *t*-test. The columns represent the following in order: Vehicle (*n* = 6–9); DSF-41 5 mg/kg (*n* = 8), 10 mg/kg (*n* = 8); cyanamide 40 mg/kg (*n* = 6); diazepam 1.5 mg/kg (*n* = 6). Statistical significance is denoted by **p < 0.01 vs. vehicle-treated mice. ns, no significant difference.

DSF significantly and dose-dependently increased the time the mice spent in the open arms 30 min after its administration [F (3, 30) = 16.64, *p* < 0.0001]. Bonferroni analysis revealed a significant effect of DSF compared with the vehicle-treated group (20 mg/kg, i. p.: *t* = 0.9894, *p* > 0.05; 40 mg/kg, i. p.: *t* = 3.863, *p* < 0.01; 80 mg/kg, i. p.: *t* = 6.417, *p* < 0.001; Figure 1A). The Student’s *t*-test analysis revealed a significant effect of diazepam (1.5 mg/kg, s. c.) compared with the vehicle-treated group (*t* = 5.038, *p* < 0.001; Figure 2E).

DSF significantly and dose-dependently increased the percentage of open arm entries [F (3, 30) = 14.24, *p* < 0.0001]. Bonferroni analysis revealed a significant effect of DSF compared with the vehicle-treated group (20 mg/kg, i. p.: *t* = 0.3999, *p* > 0.05; 40 mg/kg, i. p.: *t* = 2.693, *p* > 0.05; 80 mg/kg, i. p.: *t* = 5.864, *p* < 0.001; Figure 1B). The Student’s *t*-test analysis revealed a significant effect of diazepam (1.5 mg/kg, s. c.) compared with the vehicle-treated group (*t* = 3.733, *p* < 0.005; Figure 2F).

As shown in Figure 1C, DSF (40 mg/kg) resulted in a significantly increased percentage of time spent in the open arms at 15, 30 and 60 min after administration [F (4, 37) = 5.626, *p* < 0.0012]. The peak effect for time spent in the open arms occurred at 30 min after DSF was administered (15 min: *t* = 2.870, *p* < 0.05; 30 min: *t* = 3.911, *p* < 0.01; 60 min: *t* = 2.883, *p* < 0.05; 120 min: *t* = 0.4859, *p* > 0.05; Figure 1C).

As shown in Figure 1D, DSF (40 mg/kg) resulted in an increased percentage of open arm entries, but the difference was not significant. The peak effect for open arm entries occurred at 30 min after DSF was administered.

Neither DSF at any dose (20–80 mg/kg) or time (15–120 min) nor diazepam (1.5 mg/kg) had a significant effect on the total number of arm entries (Table 2 and Table 3), suggesting that there were no apparent sedative effects at the dosages used.

**TABLE 2:** Effects of DSF, cyanamide, DSF-41, and diazepam on the total number of arm entries in the mouse elevated plus-maze test.

Drugs	Dose (mg/kg)	Number of animals	Total arm entries
DSF	Vehicle	9	22.8 ± 1.5
	20	8	22.5 ± 1.6
	40	8	23.1 ± 1.2
	80	9	21.7 ± 1.6
Cyanamide	Vehicle	6	24.8 ± 1.4
	40	6	26.2 ± 0.9
DSF-41	Vehicle	8	25.1 ± 1.5
	5	8	19.1 ± 1.5*
	10	8	21.6 ± 1.4
Diazepam	Vehicle	6	20.2 ± 2.0
	1.5	6	27.0 ± 4.4

DSF (20, 40, or 80 mg/kg, i.p.); cyanamide (40 mg/kg); DSF-41 (5 or 10 mg/kg); and vehicle were administered 30 min before the test. Data represent the means ± SEM., Statistical significance is denoted by *p < 0.05 vs. vehicle-treated mice.

**TABLE 3:** Effects of DSF (40 mg/kg, i.p.) on the total number of arm entries in the mouse elevated plus-maze test.

Drug (dose)	Time (min)	Number of animals	Total arm entries
DSF (40 mg/kg)	Vehicle	8	22.4 ± 1.6
	15	9	23.1 ± 1.1
	30	8	23.1 ± 1.2
	60	8	25.1 ± 1.4
	120	8	22.1 ± 1.3

DSF (40 mg/kg, i.p.) was administered 15, 30, 60, or 120 min before the test. Vehicle (i.p.) was administered 30 min before the test. Data represent the means ± SEM.

#### 3.1.2 The Effect of DSF-41 on the Percentage of Time Spent in and Entries to Open Arms

DSF-41, a potent FROUNT inhibitor, significantly and dose-dependently increased the time mice spent in the open arms 30 min after administration [F (2, 21) = 7.353, *p* = 0.0038]. Bonferroni analysis revealed a significant effect of DSF-41 compared with the vehicle-treated group (5 mg/kg, i. p.: *t* = 1.264, *p* > 0.05; 10 mg/kg, i. p.: *t* = 3.767, *p* < 0.01; Figure 2A).

DSF-41 also significantly and dose-dependently increased the percentage of open arm entries [F (2, 21) = 10.63, *p* = 0.0006]. Bonferroni analysis revealed a significant effect of DSF-41 compared with the vehicle-treated group (5 mg/kg, i. p.: *t* = 1.824, *p* > 0.05; 10 mg/kg, i. p.: *t* = 4.580, *p* < 0.001; Figure 2B). As shown in Table 2, DSF-41 (5 mg/kg, *t* = 2.822, *p* < 0.05) significantly decreased the total number of arm entries. However, at a higher concentration, DSF-41 (10 mg/kg, *t* = 1.646 *p* > 0.05) had no significant effect on total arm entries (Table 2).

#### 3.1.3 The Effect of Cyanamide on the Percentage of Time Spent in and Entries to Open Arms

Cyanamide (40 mg/kg), another ALDH inhibitor, had no significant effect on the percentage of time spent in (*t* = 0.9652, *p* > 0.05; Figure 2C) and entries into (*t* = 0.6066, *p* > 0.05; Figure 2D) the open arms 30 min after administration (*t* = 0.9652, *p* > 0.05; Figure 2C).

Cyanamide (40 mg/kg) had no significant effect on the total number of arm entries compared with the vehicle (Table 2).

### 3.2 Measurement of Spontaneous Locomotor Activity

**FIGURE 3:**
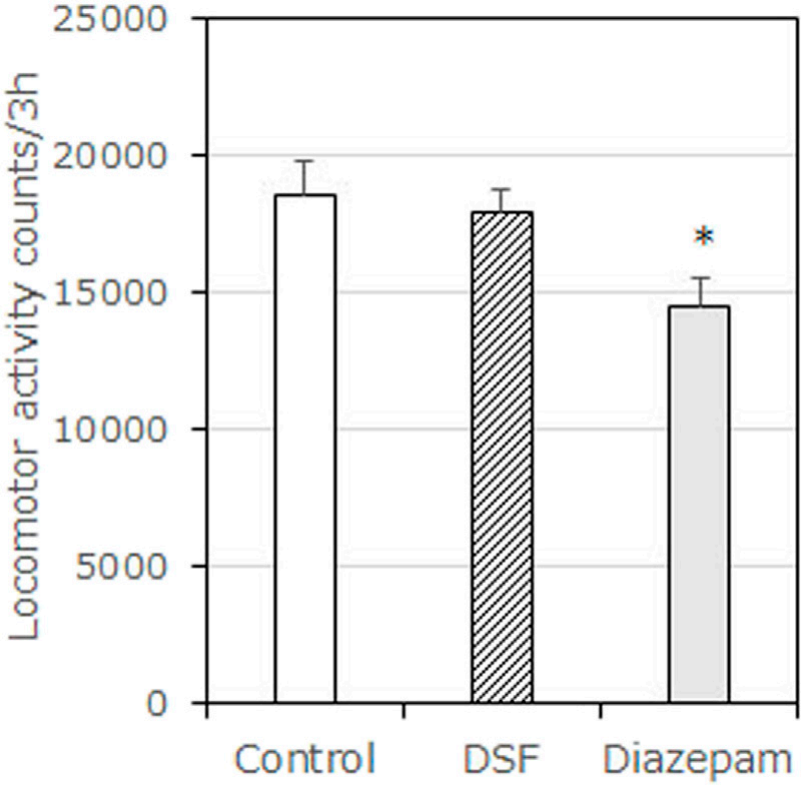
Effects of DSF on locomotor activity test in mice. Each column represents the mean total locomotor counts ±SEM for 210 min after administration. Diazepam was used as a positive control anxiolytic drug. DSF (80 mg/kg, i. p.) and diazepam (1.5 mg/kg, s. c.) were administered 30 min before the test. The statistical significance of differences was assessed with one-way ANOVA. Post-hoc individual group comparisons were made with Bonferroni’s test. The columns represent the following in order: Vehicle (*n* = 9); DSF 80 mg/kg (*n* = 6); diazepam 1.5 mg/kg (*n* = 10). Statistical significance is denoted by *p < 0.05 vs. vehicle-treated mice.

DSF (80 mg/kg) had no significant effect on spontaneous locomotor activity compared with the vehicle (Figure 3). However, diazepam (1.5 mg/kg) significantly decreased spontaneous locomotor activity [F (2, 22) = 8.543, *p* = 0.0018]. Bonferroni analysis revealed a significant effect of diazepam compared with the vehicle-treated group (1.5 mg/kg, s. c.: *t* = 4.016, *p* < 0.01; Figure 3).

### 3.3 Y-Maze Test

**FIGURE 4:**
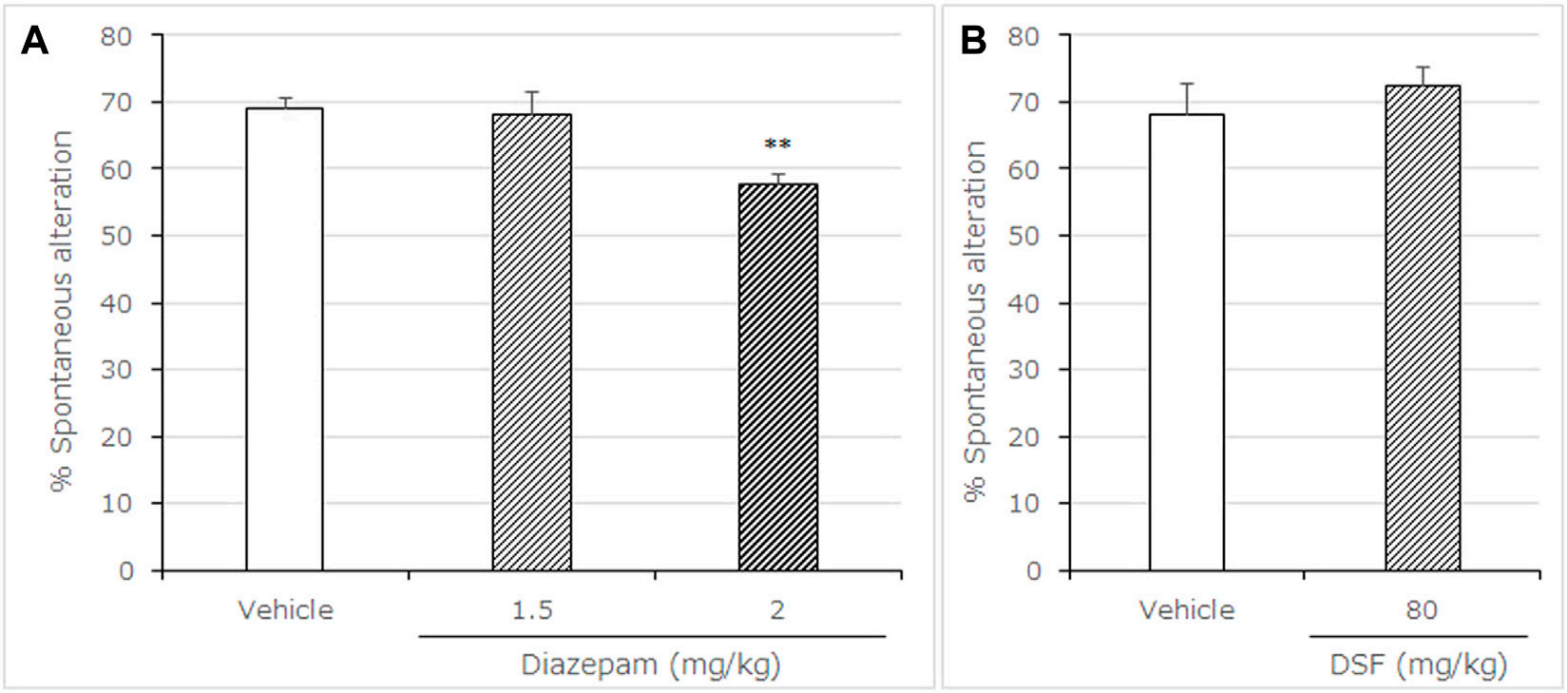
Effects of DSF on mice in the Y-maze test. Panel **(A)** shows the percentage of spontaneous alternation. Panel **(B)** shows the total arm entries. Diazepam was used as a positive control anxiolytic drug. DSF (80 mg/kg, i. p.), diazepam (1.5, 2.0 mg/kg, s. c.), and vehicle were administered 30 min before the test. Each column represents the mean ± SEM. The statistical significance of differences among drugs was assessed using one-way ANOVA. Post-hoc individual group comparisons were made with Bonferroni’s test. The columns represent the following in order: Vehicle (*n* = 11); DSF 80 mg/kg (*n* = 6); diazepam 1.5 mg/kg (*n* = 7), 2.0 mg/kg (*n* = 9). Statistical significance is denoted by **p < 0.01 vs. vehicle-treated mice. ns, no significant difference.

Diazepam (2.0 mg/kg, s. c.) significantly decreased the percentage of spontaneous alternations [F (2, 22) = 9.675, *p* = 0.0010; Bonferroni analysis *t* = 3.531, *p* < 0.01; Figure 4A]. In contrast to diazepam, DSF (80 mg/kg, i. p.) caused no significant changes in the percentage of spontaneous alternation behavior compared with the vehicle (*t* = 0.8145, *p* > 0.05; Figure 4B). In addition, neither DSF (80 mg/kg, s. c.) nor diazepam (2.0 mg/kg, s. c.) significantly affected total arm entries (Table 4), suggesting that there were no apparent sedative effects at the dosages used.

**TABLE 4:** Effects of DSF and diazepam on the total number of arm entries in the mouse Y-maze test.

Drugs	Dose (mg/kg)	Number of animals	Total arm entries
Diazepam	Vehicle	8	46.8 ± 2.8
	1.5	7	58.3 ± 6.3
	2.0	9	34.3 ± 3.0
DSF	Vehicle	6	35.5 ± 2.7
	80	6	32.2 ± 3.2

Diazepam (1.5 or 2.0 mg/kg, s.c.); DSF-41 (80 mg/kg, i.p.); and vehicle were administered 30 min before the test. Data represent the means ± SEM., Statistical significance is denoted by *p < 0.05 vs. vehicle-treated mice.

### 3.4 Rotarod Test

**FIGURE 5:**
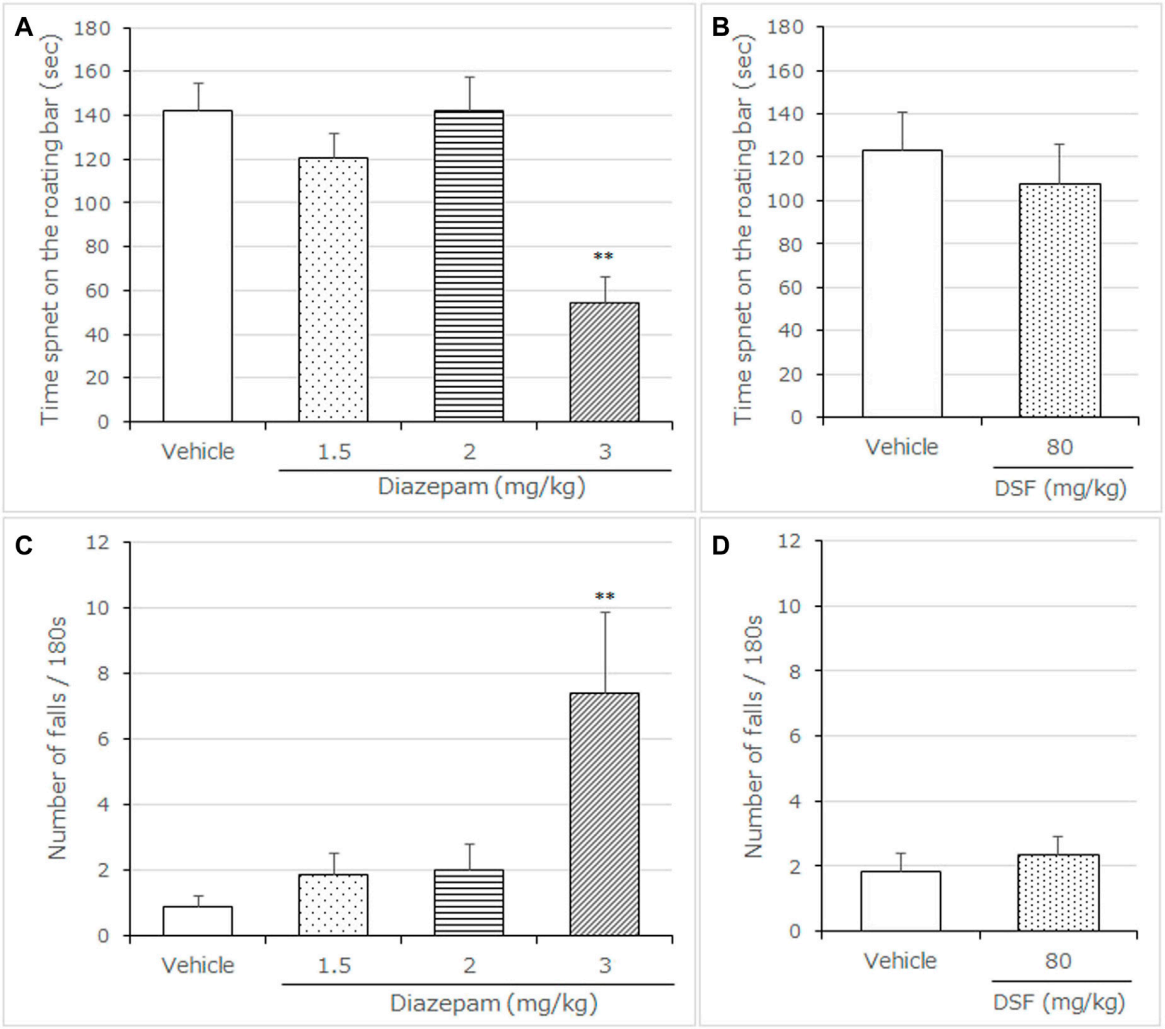
Effects of DSF on mice in the rotarod test. Panel **(A,B)** shows the maximum time spent on the rotating bar. Panel **(C,D)** shows the number of falls. Diazepam was used as a positive control anxiolytic drug. DSF (80 mg/kg, i. p.), diazepam (3.0 mg/kg, s. c.), and vehicle were administered 30 min before the test. Each column represents the mean ± SEM. The statistical analysis was performed using Student’s *t*-test. The columns represent the following in order: Vehicle (*n* = 6); DSF 80 mg/kg (*n* = 6); diazepam 3.0 mg/kg (*n* = 5). Statistical significance is denoted by *p < 0.05 and **p < 0.01, respectively, vs. vehicle-treated mice. ns, no significant difference.

Diazepam (3.0 mg/kg, s. c.) significantly decreased the maximum time spent on the rotarod compared with the vehicle-treated group ((F (3, 21) = 8.472, *p* = 0.0007, Bonferroni analysis *t* = 4.709, Figure 5A). In contrast to diazepam, DSF (80 mg/kg, i. p.) caused no significant changes in the maximum time spent on the rotarod compared with the vehicle-treated group (*t* = 0.556, *p* > 0.05; Figure 5B). In addition, diazepam (3.0 mg/kg, s. c.) significantly increased the number of falls from the rotarod [(F (3, 21) = 6.902, *p* = 0.0021, *t* = 4.384, Figure 5C], while DSF (80 mg/kg, i. p.) caused no significant changes to the number of falls from the rotarod compared with the vehicle (*t* = 0.5350, *p* > 0.05; Figure 5D).

### 3.5 Noradrenaline and its Metabolites Levels

DSF is known to have a weak inhibitory effect on dopamine *β*-hydroxylase (DBH), which catalyzes the conversion of dopamine to norepinephrine (Karamanakos et al., 2001; Bourdélat-Parks et al., 2005). Therefore, we quantified the levels of noradrenalin and its associated metabolite (MHPG) in four brain regions: the amygdala, hypothalamus, PFC, and striatum.

The selective DBH inhibitor fusaric acid (FA) was used as positive control (Vetulani et al., 1973). FA (100 mg/kg) significantly decreased the noradrenaline contents in all of the brain regions as follows: amygdala (*t* = 7.027, *p* < 0.0001), hypothalamus (*t* = 4.219, *p* < 0.001), PFC (*t* = 4.010, *p* < 0.005), and striatum (*t* = 2.980, *p* < 0.01) (Supplementary Table S1). However, DSF (80 mg/kg) did not affect the noradrenaline contents in the four brain regions (Table 5).

**TABLE 5:** Effects of DSF on norepinephrine and its metabolite MHPG in the prefrontal cortex, hypothalamus, amygdala, and striatum of mice.

Brain region	Group	Dose	ng/mg wet tissue	
			NE	MHPG
Frontal cortex	Vehicle	0	0.560 ± 0.030	4.260 ± 0.267
	DSF	80	0.508 ± 0.021	4.841 ± 0.280
Hypothalamus	Vehicle	0	2.558 ± 0.389	6.590 ± 0.537
	DSF	80	2.680 ± 0.426	8.877 ± 1.299
Amygdala	Vehicle	0	0.066 ± 0.003	0.534 ± 0.026
	DSF	80	0.056 ± 0.003	0.604 ± 0.029
Striatum	Vehicle	0	0.143 ± 0.021	7.111 ± 0.439
	DSF	80	0.179 ± 0.029	7.145 ± 0.484

DSF (80 mg/kg, i.p.) and vehicle were administered 30 min before the test. Data represent the means ± SEM., Vehicle (*n* = 4); DSF, 80 mg/kg (*n* = 8). Statistical significance is denoted by *p < 0.05 vs. vehicle-treated mice. NE, norepinephrine.

### 3.6 Glutamate Levels of PL-PFC After Exposure to Stress in DSF-Treated Mice

**FIGURE 6:**
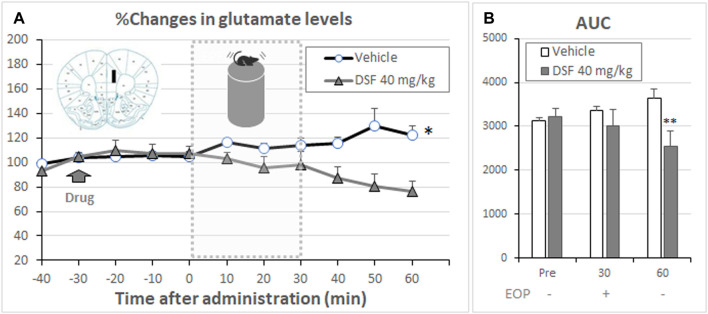
Effect of DSF on extracellular glutamate levels. Panel **(A)** shows the percentage change in the extracellular levels of glutamate in the PL-PFC. After stabilization of the extracellular glutamate levels, DSF (40 mg/kg, i. p.) was administered. The mice were exposed to the elevated open-platform stress 30 min after drug administration and then returned to the cage (one cage per animal) following 30 min of exposure to stress. Dialysate samples were collected for 10 min. Glutamate levels are expressed as a percentage of the basal levels, which were calculated from four consecutive samples before drug administration. The data represent the mean ± SEM (*n* = 8–11 mice/group). *p < 0.05 vs. Base line data at 0 min (two-way ANOVA followed by Bonferroni’s test). The AUC (% min) for extracellular glutamate [Panel **(B)**] levels in the PL-PFC was calculated from the respective time-course curves over a period of 30 min after DSF administration (Pre). Each column represents the mean ± SEM (*n* = 8–11 mice/group). **p < 0.01 vs. vehicle group (two-way ANOVA followed by Bonferroni’s test).

The glutamate levels in PL-PFC play an important role in the development of anxiety-like behavior in mice (Saitoh et al., 2014; Ohashi et al., 2015; Suzuki et al., 2016; Saitoh et al., 2017; Saitoh et al., 2018a). Therefore, we examined the effect of extracellular glutamate levels in the DSF-treated mice during stress exposure on the elevated open-platform. The percentage changes in extracellular glutamate levels of the PL-PFC were increased following exposure to the elevated open-platform stress for 30 min in vehicle-treated mice (F (6,108) = 1.56, *p* = 0.1659; Figure 6A). Statistically significant increases from the average baseline levels were detected at 50 min in the vehicle-treated group (Bonferroni’s test, *t* = 2.964, *p* < 0.05). However, DSF- (40 mg/kg) treated mice exhibited no changes in the extracellular glutamate levels both during and following the period of stress. The levels were not statistically different from the average of their baseline levels. The AUC (%·min) for extracellular glutamate in the PL-PFC showed a significant decrease in the DSF-treated groups compared with the vehicle-treated group (F (2,34) = 126.5, *p* < 0.0001, Bonferroni’s test, *t* = 13.94, *p* < 0.01; Figure 6B).

## 4 Discussion

### 4.1 The Anxiolytic-Like Effects of DSF in the Elevated Plus-Maze Test

In this study, DSF resulted in significant dose- and a time-dependent increases in the time spent by mice in the open arms of the maze during an EPM test. The peak effects of DSF (40 mg/kg) 30 min after intraperitoneal administration were same as the typical effects of the benzodiazepine anxiolytic diazepam. Thus, we suggest that DSF produces potent anxiolytic-like effects in mice. This is the first report on the anxiolytic-like effects of DSF in rodents.

We recently discovered that DSF specifically and potently inhibits the interaction between FROUNT and both CCR2 and CCR5, which share a FROUNT-binding element (Toda et al., 2014; Terashima et al., 2020). Therefore, we hypothesized that the inhibitory effects of FROUNT are involved in the anxiolytic-like effects of DSF. To clarify this hypothesis, we synthesized a novel compound, DSF-41, which suppresses the FROUNT signal more strongly than DSF (Table 1). Interestingly, DSF-41 produced the anxiolytic-like effects at doses four times lower than those required for DSF to produce the same effects, suggesting that FROUNT inhibition produced the anxiolytic-like effects seen in EPM test.

Conversely, DSF also inhibits ALDH. However, the selective and more potent ALDH inhibitor cyanamide, which inhibits neither FROUNT nor CCR2 (Table 1) had no effect on the results of the EPM test. These results are consistent with those of a previous report on cyanamide using a mouse EPM test (Tambour et al., 2005). The cyanamide dosage used (40 mg/kg, i. p.) has been shown to increase blood and brain acetaldehyde levels (Jamal et al., 2003). Therefore, we suggest that ALDH activity was not involved in the mechanism underlying the anxiolytic-like effects of DSF.

Taken together, we propose that the suppression of FROUNT signaling may play a role in the anxiolytic-like effects of DSF.

DSF is sparingly soluble in aqueous buffers. Thus, based on the results of previous studies (Hubbard et al., 2017; Terashima et al., 2020), corn oil was used as a solvent in the present study to dissolve DSF at a maximum concentration of 8 mg/ml. The absorption of DSF may have been lower because the abdominal area is an aqueous environment. Future studies using DSF derivatives with higher solubility in aqueous buffers will be needed to evaluate the activity of the FROUNT inhibitor.

### 4.2 The Mechanisms of the Anxiolytic-Like Effects Induced by DSF

Using microanalysis, we determined previously that increased extracellular glutamate levels in the PL-PFC of mice produce anxiety-like behaviors (Saitoh et al., 2014; Suzuki et al., 2016), and that these anxiety-like behaviors are diminished by treatment with several anxiolytics, in company with the attenuation of glutamate levels in the PL-PFC (Ohashi et al., 2015; Saitoh et al., 2018a; Saitoh et al., 2018b). Thus, we suggested that the activation of glutamate transmission in the PL-PFC plays important roles in the expression of anxiety-like behaviors in mice (Saitoh et al., 2014; Suzuki et al., 2016; Nagase and Saitoh, 2020). In the present study, we found that the extracellular glutamate levels in PL-PFC significantly increased during stress exposure on the elevated open-platform, which is known as a psychological stress model in rodents (Miyata et al., 2007a; Miyata et al., 2007b). Interestingly, DSF significantly and completely attenuated the increased glutamate levels in the PL-PFC. Several studies have suggested that chemokine receptors colocalize with GABAergic neurons and control GABA release. Previously, it was reported that CCL2 causes a reduction in GABA-induced transient inward currents (Gosselin et al., 2005), while another group indicated that treatment with the chemokine receptor analog SAM2 increases the frequency of spontaneous inhibitory postsynaptic currents (Choi et al., 2018). These results suggested that chemokine receptors inhibit GABAergic neurons through neuromodulation. Taken together, we suggest that the inhibitory effects on chemokine signaling induced by DSF may increase GABA release, as excitatory glutamate transmission was found to be attenuated in the PL-PFC. We therefore proposed that DSF produces anxiolytic-like effects by the presynaptic suppression of activated glutamatergic transmission in the PL-PFC.

DSF is also known to have a weak inhibitory effect on the enzyme DBH, which catalyzes the conversion of dopamine to norepinephrine in the brain. The selective DBH inhibitor FA is known to reduce the content of noradrenalin in rodent brains (Vetulani et al., 1973). In this study, we found that there were no significant effects on the levels of noradrenaline and its metabolites 30 min after DSF (80 mg/kg, i. p.) treatment in the PFC, amygdala, striatum, and midbrain, which are brain areas responsible for the modulation of emotional behaviors, although FA (100 mg/kg, i. p.) significantly decreased the levels of noradrenaline and its metabolites in these areas as previously reported (Vetulani et al., 1973). These results suggested that DSF at the dose used in the present study had no effects on the enzyme DBH activity in the areas of the brain responsible for the modulation of emotional behaviors.

On the other hand, previous studies have reported that DSF significantly decreased the noradrenaline contents in these brain regions. For example, Bourdélat-Parks et al. indicated that the administration DSF (50–200 mg/kg) every 2 h significantly decreased the noradrenaline contents of PFC in rats (Bourdélat-Parks et al., 2005). Moreover, another report indicated that continuous administration of a higher dose of DSF (100 mg/kg, i. p.) for 5 days significantly decreased the noradrenaline contents of PFC in rats (Karamanakos et al., 2001). Indeed, continual rather than single treatments with DSF may be required to cause an effect on noradrenaline levels in rodent brains. Taken together, we suggest that the anxiolytic-like effects of DSF are not much associated with its capacity to inhibit DBH.

### 4.3 Effects of DSF on the Benzodiazepine-Related Adverse Effects

The GABA_A_ receptor, an active site for benzodiazepines, plays an important role in the pathophysiology of the adverse effects associated with these drugs, which include motor coordination deficits, muscle weakness, sedation, and amnesia.

The rotarod test was performed to evaluate motor coordination deficits, muscle weakness, and sedation in mice after DSF or diazepam treatment (Talarek et al., 2008; Razavi et al., 2017; Chen et al., 2018). This type of analysis is well established and widely used for measuring motor coordination, muscle weakness, and sedation in rodents, by observing the length of time spent on the rotating rod and the number of falls (Chen et al., 2018). In addition to its roles described above, the GABA_A_ receptor is important in the pathophysiology of these motor coordination deficits (Razavi et al., 2017). In this study we found that mice treated with diazepam at a dose that was twice that which produces anxiolytic-like effects reduced the time spent on the rotating rod and increased the number of falls recorded, indicating that there was a motor impairment effect that influenced motor coordination and led to muscle weakness. Similar results were obtained in a previous study (Talarek et al., 2008; Talarek et al., 2010; Chen et al., 2018). Contrary to diazepam, DSF at the doses required for anxiolytic-like effects did not significantly influence motor coordination in the rotarod test. We therefore suggest that DSF does not act on the rat brain region(s) responsible for motor coordination. Furthermore, DSF did not affect the total number of arm entries in the EPM and Y-maze tests. Therefore, at doses that cause anxiolytic-like effects, DSF produced neither motor coordination deficits nor oversedation.

In the present study, we showed that DSF produced no amnesic effects at the dose required for anxiolytic-like effects in the Y-maze test. Amnesia is a classical side effect of benzodiazepines and is seen with sub-ataxic doses in both humans (Lister, 1985) and animals (Thiébot, 1985). It has been reported that diazepam decreases spontaneous alternation in the Y-maze test. We previously suggested that diazepam at the dose required for an anxiolytic effect produced amnesic effects in a rat Y-maze test (Sugiyama et al., 2012; Saitoh et al., 2013). In the present study, we also obtained similar results in mice treated with a 1.3 times higher dose of diazepam than that shown to produce anxiolytic-like effects. In contrast, DSF had no effects on spontaneous alternation in the Y-maze test at more than twice the dose needed to produce anxiolytic-like effects. Our results suggest that DSF, at doses that produce anxiolytic effects, does not affect the GABA_A_-benzodiazepine receptor complex in the mouse brain regions responsible for benzodiazepine-associated side effects.

A previous study reported that diazepam caused amnesia at a lower dose (1 mg/kg) in a modified EPM task (Orzelska et al., 2013) as compared with the dose of diazepam (2 mg/kg), at which amnesia was observed in our study. In the present study, 6–10 week old ICR mice were used for the Y-maze test, whereas Orzelska et al. used 3–5 week old Swiss mice (20–25 g body weight). It is possible that the differences in the age and strain of the mice used could have affected the results.

In the present study, 1.5 mg/kg of diazepam led to disturbances in locomotion during the measurement of spontaneous locomotor activity, while a higher dose (2.0 mg/kg) of diazepam in the EPM and Y-maze tests did not affect the total number of arm entry. The shorter analysis time in the EPM and Y-maze tests as compared with that in the spontaneous locomotor activity test was considered to be the reason why no effect on extrapolatory behavior was observed. The EPM and Y-maze tests were performed for 5 and 8 min, respectively, whereas the spontaneous locomotor activity test was performed for 3 h.

### 4.4 Future Development of DSF as a Candidate Novel Therapeutic Agent

It has been well established that a significant proportion of patients with mood and anxiety-related disorders exhibit evidence of elevated inflammatory markers, including increases in cerebrospinal fluid and circulating concentrations of inflammatory chemokines and cytokines (Eyre et al., 2016; Felger, 2018). A previous clinical study indicated that DSF was more effective than a placebo and as effective as the benzodiazepine anxiolytic chlordiazepoxide at reducing anxiety in alcoholic outpatients (Rosenberg, 1974). Another study indicated that coadministration of DSF and lorazepam to alcoholic patients with co-occurring anxiety disorder largely reduced anxiety, depression, and cravings (Bogenschutz et al., 2016). However, other investigations reported no statistically significant anxiolytic effects attributable to DSF in alcoholics (Snyder and Keeler, 1981; Goyer et al., 1984). These discrepancies might be attributed to the different methods used to assess mood changes. Nevertheless, the plasma DBH activity in alcoholic patients was found to be significantly lower than that observed in healthy controls (Köhnke et al., 2002). As mentioned above, DSF is inhibitory to the enzyme DBH. Therefore, the patients with lower DBH activity might be more likely to develop a reaction to psychoactive symptoms induced by the inhibitory effect of DSF on DBH, which occur rarely with higher doses of DSF (Mohapatra and Rath, 2017). Further clinical studies are needed to clarify the effects of DSF on anxiety disorders. We propose that DSF is a candidate novel therapeutic agent for the improved treatment of mood and anxiety-related disorders in patients who suffer from high levels of inflammation.

## 5 Conclusion

This is the first report on the anxiolytic-like effects of DSF in rodents. In this study, we found that DSF, which potently inhibits the cytoplasmic protein FROUNT, produces potent anxiolytic-like effects without triggering diazepam-related adverse effects, such as motor coordination deficits, oversedation, and amnesia. Moreover, we propose that DSF produces the anxiolytic-like effects through the presynaptic suppression of activated glutamatergic transmission in the PL-PFC. Furthermore, we demonstrated that, although the ALDH inhibitor cyanamide produced no anxiolytic-like effects, the more potent and selective compound DSF-41 produced robust anxiolytic-like effects. Taken together, we propose that the inhibition of FROUNT function by DSF provides an effective therapeutic option in anxiety, focusing on intracellular chemokine-FROUNT signaling molecules.

## Data Availability

The raw data supporting the conclusion of this article will be made available by the authors, without undue reservation.
