# Supplementary material for: Disulfiram Produces Potent Anxiolytic-Like Effects Without Benzodiazepine Anxiolytics-Related Adverse Effects in Mice
Source: Front Pharmacol. 2022 Mar 7;13:826783. doi: 10.3389/fphar.2022.826783 (PMC8940232; doi:10.3389/fphar.2022.826783)
Supplement: Supplementary file 1 [file Table1.DOCX]

**Supplemental table**

Table S1

Effects of fusaric acid on NE and its metabolite MHPG in the mice frontal cortex, hypothalamus amygdala and striatum.

| Brain region | Group | Dose | ng/mg wet tissue | |
| --- | --- | --- | --- | --- |
|  |  |  | NE | MHPG |
| Frontal cortex | Vehicle | 0 | 0.819±0.072 | 0.440±0.026 |
|  | FA | 100 | 0.438±0.063* | 0.709±0.094* |
| Hypothalamus | Vehicle | 0 | 3.196±0.282 | 0.703±0.077 |
|  | FA | 100 | 1.729±0.203* | 0.939±0.143 |
| Amygdala | Vehicle | 0 | 0.850±0.043 | 0.505±0.048 |
|  | FA | 100 | 0.334±0.060* | 0.705±0.088 |
| Striatum | Vehicle | 0 | 0.274±0.024 | 0.240±0.074 |
|  | FA | 100 | 0.169±0.026* | 0.662±0.111* |

Fusaric acid (FA; 100 mg/kg, i.p.) and vehicle were administered 180 min before the test. Data represent means ± SEM. Vehicle (n = 8); Fusaric acid 100 mg/kg (n = 8). Statistical significance is denoted by * (p < 0.01, vs. vehicle-treated mice).
